# Connecting metabolome and phenotype: recent advances in functional metabolomics tools for the identification of bioactive natural products

**DOI:** 10.1039/d3np00050h

**Published:** 2024-02-14

**Authors:** Giovanni Andrea Vitale, Christian Geibel, Vidit Minda, Mingxun Wang, Allegra T. Aron, Daniel Petras

**Affiliations:** a CMFI Cluster of Excellence, Interfaculty Institute of Microbiology and Medicine, University of Tuebingen Tuebingen Germany; b Division of Pharmacology and Pharmaceutical Sciences, University of Missouri – Kansas City Kansas City USA; c Department of Computer Science, University of California Riverside Riverside USA mingxun.wang@ucr.edu; d Department of Chemistry and Biochemistry, University of Denver Denver USA allegra.aron@du.edu; e Department of Biochemistry, University of California Riverside Riverside USA dpetras@ucr.edu

## Abstract

Covering: 1995 to 2023

Advances in bioanalytical methods, particularly mass spectrometry, have provided valuable molecular insights into the mechanisms of life. Non-targeted metabolomics aims to detect and (relatively) quantify all observable small molecules present in a biological system. By comparing small molecule abundances between different conditions or timepoints in a biological system, researchers can generate new hypotheses and begin to understand causes of observed phenotypes. Functional metabolomics aims to investigate the functional roles of metabolites at the scale of the metabolome. However, most functional metabolomics studies rely on indirect measurements and correlation analyses, which leads to ambiguity in the precise definition of functional metabolomics. In contrast, the field of natural products has a history of identifying the structures and bioactivities of primary and specialized metabolites. Here, we propose to expand and reframe functional metabolomics by integrating concepts from the fields of natural products and chemical biology. We highlight emerging functional metabolomics approaches that shift the focus from correlation to physical interactions, and we discuss how this allows researchers to uncover causal relationships between molecules and phenotypes.

## Abstract 2

In this review we discuss emerging functional metabolomics strategies and their potential use to reveal mechanistic insights in large-scale natural product discovery studies.

## Graphical abstract

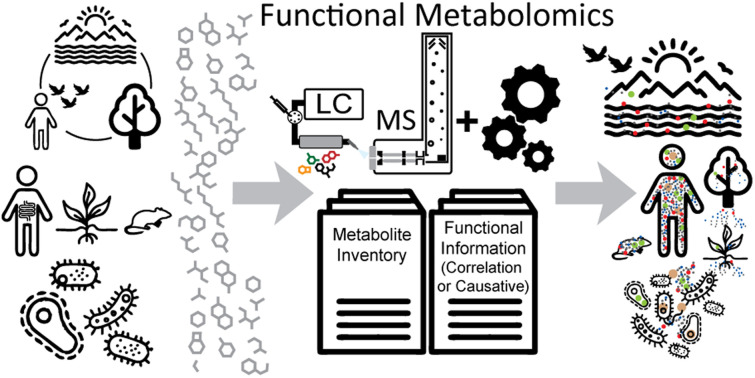

## 1. Introduction

Advances in bioanalytical methods have yielded fascinating molecular insights into the mechanisms of life. Within this myriad of analytical approaches, mass spectrometry represents one of the most sensitive and selective techniques that can provide molecular inventories from mega-Dalton protein complexes to small metabolites. Creation of molecular inventories *via* mass spectrometry technologies thereby ranges from planetary scales to single-cell resolution.^1–4^

**Fig. 1:**
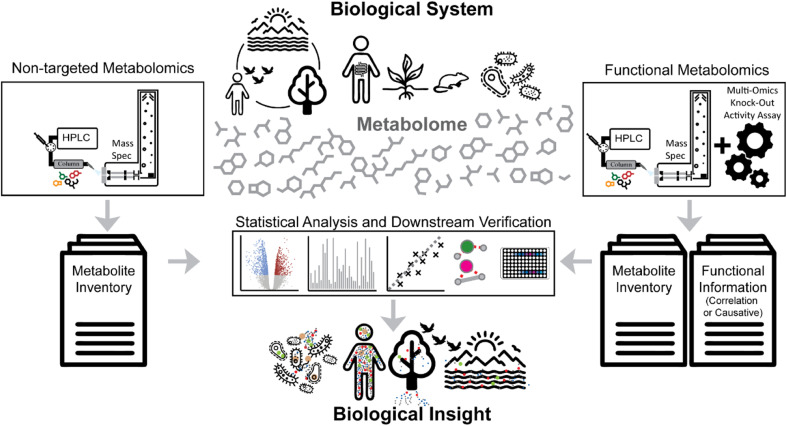
Metabolomics strategies aim to provide biological insights at the molecular level. Non-targeted metabolomics (left side) aims to provide inventories of (ideally) all metabolites in a given sample and compare them between different biological stages. Functional metabolomics approaches (right side) aim to add functional associations (*e.g.*, genes) or direct causative relations between particular metabolites and biological functions (*e.g.*, bioactivity or enzyme inhibition).

Non-targeted metabolomics focuses on analyzing all small molecules in a given biological system, including endogenous and exogenous metabolites, peptides, and anthropogenic compounds (*e.g.*, synthetic drugs, pesticides, and other artificial chemicals) (Fig. 1, left side). Such experiments aim to identify and quantify all small molecules under different conditions or stages of the biological system of interest, akin to comparative genomics and transcriptomics studies. For example, a non-targeted metabolomics experiment could compare two groups of animals - one healthy and one disease model – to better understand the disease phenotype. Using statistical analysis to compare the metabolic differences between groups or the metabolic changes in a time course study not only highlights potential biomarkers and molecules of interest but also helps generate new hypotheses.

The functional genomics community has aimed to define the role of genes and their products in an observed phenotype at the organism scale.^5^ This strategy has successfully uncovered the genetic causes of various diseases and other cellular regulation mechanisms.^6–8^ Similarly, the field of metabolomics has adapted strategies to correlate metabolic responses to individual genes and their regulation, often described as “functional metabolomics”.^9,10^ In a broader sense, functional metabolomics, sometimes also referred to as “activity metabolomics”,^11^ aims to investigate the functional roles of metabolites at the scale of the metabolome.^12^ Yet, most functional metabolomics studies make use of indirect measurements of functional properties of metabolites; for example, by inferring interaction through gene deletion mutants, knockdowns, or other perturbations and computational enrichment analysis (Fig. 1, right side).^13–15^

**Fig. 2:**
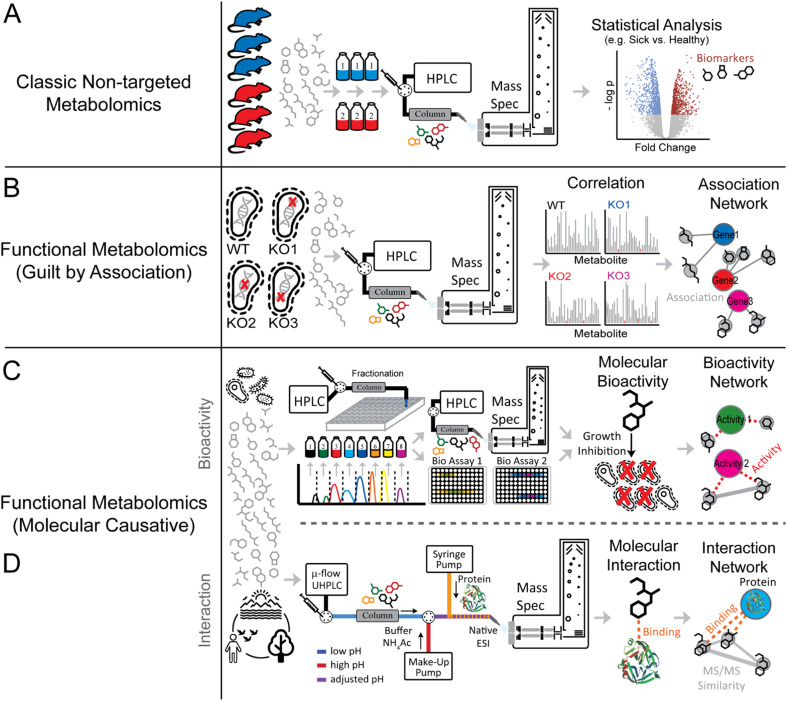
Metabolomics and functional metabolomics concept. Panels A–D highlight “classic” non-targeted metabolomics and functional metabolomics approaches. While “classic” non-targeted metabolomics approaches (Panel A) typically assess a molecular driver between different biological conditions (*e.g.*, sick *vs.* healthy), functional metabolomics approaches (Panel B) make use of systematic perturbations (*e.g.*, genome-wide knockout studies) to associate metabolites to specific genes. In addition to purely association-based insights, molecular causative functional metabolomics approaches (Panel C and D) aim to measure direct biological or biophysical properties (*e.g.*, antimicrobial activity or protein binding).

The definition of “functional metabolomics” is in some cases vaguely defined, as many workflows described as functional metabolomics strategies are nearly identical to regular non-targeted metabolomics studies where typically two or more groups of treatments or disease stages are compared (Fig. 2A). Many functional metabolomics studies do not measure functional properties of the metabolites themselves, but rather determine function using correlation and “guilt by association” with other known bioactive metabolites, transcription, or protein expression (Fig. 2B).^16–18^

The field of natural products, on the other hand, has a long-standing history of identifying both structures and bioactivities of primary and specialized metabolites (often referred to as “natural products”). Natural product discovery workflows typically include initial compound purification, followed by full structure elucidation using high resolution mass spectrometry (MS), tandem mass spectrometry (MS/MS), nuclear magnetic resonance spectroscopy (NMR), ultraviolet (UV) and infrared (IR) spectroscopy along with chemical derivatization strategies. Structure elucidation is usually followed by detailed bioactivity studies such as phenotyping with reporter cells, higher organisms, and molecular interaction studies with target proteins or other biomolecules. These studies make use of different biophysical or chemical techniques that measure direct molecular interactions, for which many comprehensive reviews exist in the literature.^11,19–21^

In this perspective, we highlight strategies that extend correlation-based functional metabolomics approaches by integrating concepts from the natural products field that enable elucidating molecular causal relationships at scale (Fig. 2C and D).^22,23^ We argue that shifting the focus from genes to molecules is a powerful way to explicitly understand how molecules and their conserved structural features affect phenotypes.^11^ Expanding the considered chemical space beyond genome scale metabolic models might be especially important as many bioactive molecules have either yet unknown biosynthesis pathways (*e.g.*, novel natural products),^24^ or may not have a biosynthetic basis at all (*e.g.*, xenobiotic molecules and their degradation products).^25^

## 2. Functional metabolomics strategies

### 2.1 Correlation and guilt by association-based approaches

A key tenet of the central dogma of molecular biology is the fact that the genome and metabolome are strictly correlated. Therefore, changes in one or more metabolites can be correlated with certain genes to facilitate uncovering gene function or detecting the origins of genetic diseases. Modern innovative genome editing technologies, such as CRISPR interference (CRISPRi), offer rapid, precise, and dependable methods to alter gene expression,^26^ and coupling these strategies with ‘omics technologies’ can yield insights into gene function or the effects of exogenous stimulus on a given organism. Mülleder and co-workers recently employed a comprehensive functional metabolomics pipeline to reveal the functions of 4913 genes in *Saccharomyces cerevisiae*.^10^ To connect these genes to specific metabolic signatures, the authors cultivated 4913 different strains each presenting a single gene deletion in minimal medium.

They found that the amino acid profile was specifically modulated by each gene deletion, indicating that comparison of each metabolic signature to phenotype perturbation provided insights into gene functions. This correlation-based functional metabolomic approach revealed the association of over 1500 genes with distinct metabolic signatures, including chromatin gene regulation along with protein translation and transport. In another study, Donati *et al.* employed the CRISPRi approach to unveil the effect of decreasing specific enzyme levels in *Escherichia coli* on both proteome and metabolome.^27^ The analysis of 30 CRISPRi *E. coli* strains revealed that through specific changes in their metabolome each strain was able to buffer enzyme level perturbations. This study supports the prevailing hypothesis of the “control theory” by demonstrating that the abundance of individual enzymes minimally influences overall metabolism because localized changes in metabolism are buffered.^28^

While the development of new metabolomics strategies has aided in the discovery of new drug candidates, the lack of high-throughput strategies for investigating mechanisms of action represents an additional bottle neck in the biodiscovery pipeline.^29,30^ To this end, Anglada-Girotto *et al.* combined gene knockdowns with metabolic profiling to develop a platform for functionally annotating the mode of action of small molecules including a set of antimicrobial and anticancer drugs against *Escherichia coli*, *Mycobacterium smegmatis*, and a human lung cancer cell line.^15^ They produced a library of metabolic signatures by silencing a several hundred genes involved in key essential biological processes *via* CRISPRi. Subsequently, they profiled the drug-induced metabolic changes of a range of small metabolites mainly human drugs. By associating gene-knockdowns to drug-induced metabolic signatures, the authors were able to *de novo* predict the modes of action of some compounds, including mechanism of action of several antimicrobial drugs. Overall, this study demonstrated that the metabolic signatures triggered by specific proteins resemble signatures induced by compounds that hit the same cellular function. This approach can be useful to find the mechanisms of action of drugs but also to expand the biological space of drug targets.

### 2.2 Bioactivity-based metabolomics approaches

Natural products (NPs) are among the most abundant and diverse sources of bioactive molecules. NPs diversity is a reflection of their evolutionary structural optimization to be selective for certain biological targets (*i.e.*, proteins or nucleic acids) to perform distinct functions, such as regulating endogenous defense mechanisms or playing roles in communication and competition with other organisms.^24^ Modern NP research has its roots in traditional medicine which uses medicinal plants, fungi, or other microorganisms as a source of natural remedies for numerous illnesses. Numerous countries and pharmaceutical companies run programs leveraging traditional medicines for the discovery of new pharmaceutical leads and treatment strategies.^31^

**Fig. 3:**
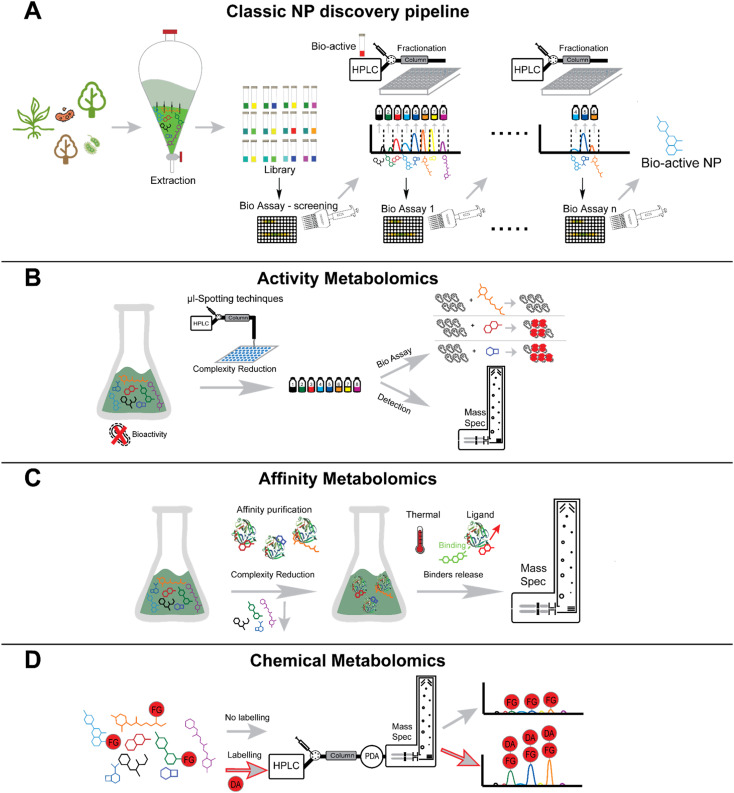
Bioactivity-guided and functional metabolomics approaches in natural products discovery. Classic NPs bioactivity-driven pipeline (Panel A), high-throughput phenotyping (Panel B), enzyme affinity screening approach (Panel C), chemical metabolomics approach (Panel D).

Although scientific advances in the fields of compound purification and characterization have enabled modern NP research, the “classical” bioactivity-guided approach still represents the most commonly employed discovery method in the NP research field.^32^ This approach intends to identify the active component(s) present in the complex natural extract containing several metabolites. The natural extracts are screened for the bioactivity of interest, for instance, antibacterial or anticancer activity. If the extract shows potential bioactivity, it undergoes a multi-step purification process. At each step, bioactivity assays are performed to orient the isolation of the pure bioactive compound(s) (Fig. 3A).

Upon isolation, the structure can be determined using techniques such as nuclear magnetic resonance (NMR) spectroscopy, MS, X-ray crystallography, circular dichroism (CD), and chemical assays (*e.g.*, Marfey's method). Bioactivity assays often detect a change in the UV-visible light absorbance or fluorescence. Examples of such assays include cell viability and proliferation assay using tetrazolium salts such as MTT and MTS, protease viability marker assay using glycylphenylalanyl-aminofluorocoumarin (GF-AFC) substrate, or ATP assay (luciferase assay).^33^ A number of assays have also been developed to discover antimicrobial compounds either from purified samples or complex extracts. These methods include the agar disk-diffusion method, broth dilution method, and thin-layer chromatography (TLC) bioautography assay, and each assay has distinct advantages and uses. While broth dilution and agar disk-diffusion methods are used to find the minimum inhibitory concentration and zone of inhibition of pure compounds respectively, TLC-bioautography assay combines the advantages of TLC separation with the direct identification of antimicrobial substances. In a TLC-bioautography assay, after the chromatographic separation, the TLC plate is sprayed with a culture containing the pathogen of interest, and pathogen viability can be assessed by colorimetric tests using tetrazolium salts.^34,35^ In addition to assays specifically for assessing antimicrobial compounds, other assays have been designed to detect enzymatic inhibition or to assess metal chelation.^36^

Despite leading to the discovery of many bioactive natural products, bioactivity-guided discovery methods, often dubbed as “top-down” approaches as compared to genome-guided “bottom-up” approaches, are believed to no longer yield new leads.^37–39^ The lack of new antimicrobials to combat antimicrobial resistance is a stark example of this situation.^40^ According to Pye *et al.*, the number of newly reported NPs exponentially grew from 1940s to the mid-1990s after which it became steady. In response to this trend (and promoted by the NCI, US), numerous high-throughput screens (HTS) have been developed.^38,41–43^ In order to boost HTS development, the NCI initiated the Natural Product Discovery program in 2018 to establish a library of more than 1 million natural extracts to be publicly accessible for HTS purposes.^44,45^

One of the main drawbacks with the “top-down” approach is the high rate of rediscovery of known NPs. A key limitation of this method is that NPs that are not responsive to the employed assay are often lost, while known bioactive compounds still give a positive hit. Therefore, this approach results in the often tedious and laborious purification and full chemical characterization of known structures with known activities. Microorganisms are a prolific source of NPs; the high rate of rediscovery is not an inherent problem itself, but can rather be largely attributed to difficulties in accessing uncultivable taxa or fully unlocking biosynthetic capabilities under laboratory conditions.^46^ For example, given that roughly 99% of known species have not been cultured in the laboratory, the rediscovery of compounds is due in part to re-exploration of well-studied taxa.

Having access to this unexplored fraction could mean increasing the explored chemical space and increasing the array of drugs.^47,48^ These challenges often overshadow any other issues associated with the bioactivity-driven approach. Different research groups developed novel approaches to combat this issue. For example, the Lewis group employed a multichannel device (*e.g.*, iChip^49^) to isolate and cultivate so far uncultured bacteria. They performed an antimicrobial-driven prioritization of over 10 000 isolated strains, facilitating the selection of the strain *Eleftheria terrae* because of its antimicrobial activity towards the human pathogen *Staphylococcus aureus*.

The bioassay-guided isolation of secreted antimicrobial metabolites culminated in the isolation of the structurally unusual depsipeptide, Teixobactin. Teixobactin exhibits a powerful inhibitory activity against *S. aureus* and *Mycobacterium tuberculosis* with no detectable resistance and acts through a unique mode of action.^50^ This study showcases that the bioactivity-guided approach is still able to yield remarkable achievements in drug discovery when applied to non-canonical taxa. We posit that addressing this challenge could uncover vast untapped biosynthetic potential to kickstart another wave of drug discovery.

Multiple studies have shown the efficient combination of molecular networking with bioassay-guided fractionation, often described as “bioactivity-based molecular networking”.^32,51,52^ These approaches combine bioactivity guided prefractionation with non-targeted LC-MS/MS analysis and subsequent molecular networking and bioactivity scoring. These approaches have been successful in guiding the isolation of novel compounds with specific activity (*e.g.*, antibacterial or antiviral). Examples include a set of novel antimicrobial gyrase inhibitors from the albicidin family from the plant pathogen *Xanthomonas albilineans* as well as 4β-deoxyphorbol esters with antiviral activity against chikungunya virus from plant extracts.^32,51^ Such approaches hold significant potential to connect chemotype and phenotype before tedious purification, however the challenges in validating activity of purified compounds remains. This strategy can be applied to various bioassays, making it valuable not only in drug discovery but also in diverse fields such as environmental toxicology and chemical ecology.

Besides applications in the field of natural product research, new technologies enable activity-based metabolomics (Fig. 3B) using micro fractionation collectors in combination with different biological readouts.^53–56^ An elegant use of such an approach, led to the discovery of small molecules that bind to the acetylcholine binding protein.^55^ Using this technology, compounds are separated *via* LC and spotted in a 1536 well plate in a working flow rate range of nL min^−1^. In a second inlet for the spotter head, the biological target and a tracer ligand can be mixed and spotted in the same wells as the ligand fraction. A post-column flow-splitter diverts the eluate to the well plate and to a mass spectrometer to assign distinct *m*/*z*-values of the fractions collected in the wells. Apart from this online method, there are also offline methods that do not directly mix the eluate and the target in flow. One such example was shown by the working group of Hamburger. They demonstrate a comparative approach using HPLC-based activity profiling. In this approach, extracts that exhibit bioactivity are separated by HPLC and are subsequently diverted post-column to a detector (usually UV and MS) and to a deep-well plate.^57,58^ The biotesting is also performed off-line by overlaying the bioactive fractions and the chromatogram, which reduces the complexity of an extract to only several possible bioactive compounds. This strategy was utilized to find compounds exhibiting anti-protozoic activity.^59,60^ While online readout-methods (where a possible reaction of a compound and the target typically takes places post-column) are feasible,^61^ offline readout-methods can be performed with fractionation collectors. Offline methods have the advantage that even compounds suffering from slow binding kinetics can be assigned.^55^ The incubation time after the fractionation spotting can be chosen freely and prolonged for the search of slow binders. A readout can be achieved choosing a proper method; for example, a fluorescence measurement can be performed.^55^

Another such technology is (single cell) multiplexed activity metabolomics – a technique in which the metabolomic pool of a source organism (like bacteria, plants, fungi or others) is separated *via* chromatography and characterized by both retention time and MS. Using this strategy, fractions are collected, dried, and then cells from a response organism, usually human cells, are added. Cells are then “barcoded” by *N*-hydroxysuccinimide functionalized dyes, treated by fluorescent antibodies, and subsequently measured by flow cytometry. By taking advantage of barcoding, the samples can be used for the determination of bioactivity and can be deconvoluted to determine the activity of each component. This technique was pioneered in 2018 (ref. 23) and has since then been used to find anti-cancer natural products.^62^

A combination of metabolomics and functional assay was proposed by Henquet *et al.*^22^ In this approach, crude extracts from chili peppers were fractionated using a liquid chromatography setup and offline tested against the calcium ion channel transient receptor potential channel vanilloid 1 (TRPV1). A biosensor for the determination of the activity of TRPV1 was developed; to do so, human cells expressing TRPV1 and a fluorescent intracellular calcium ion reporter were used for the monitoring of the TRPV1 activity of the fractions eluting from the column by their fluorescence signal. The change in concentration of intracellular calcium which is triggered by the capsaicin-activated TRPV1 is in this way visualized by the fluorescence arising from the intracellular calcium ion reporter. In this experiment, the authors found molecules acting as agonists of TRPV1. Furthermore, they suggest a translation of the shown experiments into an online functional metabolomics method with simultaneous UV-Vis, MS and activity (fluorescent) readout. In this, living cells will be added post-column to the eluate from the HPLC. The mobile phase has to be diluted to prevent the cells from getting in contact with high concentrations of toxic solvents that are usually used in RP chromatography, like methanol or acetonitrile. The cells that now come into contact with the eluent from the HPLC can be monitored by an online flow cell to derive information on the bioactivity. If exhibiting an affinity towards TRPV1 (and by this changing the intracellular calcium concentration), a fluorescence can be detected by the flow-through cell.

All the examples mentioned above show in our opinion promising bioactivity-based metabolomic approaches. Different methods are highlighted to show the variety and versatility of approaches that can be chosen to detect bioactivity.

### 2.3 Metabolic flux analysis and stable isotope labeling

The knowledge of the genome, transcriptome, or proteome is not sufficient to predict the metabolic state of an organism that would translate to the phenotype. Cells, and organisms at large, have a remarkable capacity to resist change in the levels of metabolites even if attempts are made to perturb the system.^63^ Instead of viewing reactions that connect the metabolites individually, considering them as a system of integrated pathways offers a better understanding of metabolism. Owing to decades of studies, metabolic maps have been developed that illustrate the network of reactions. Metabolomics experiments that only provide the concentration of the biomolecules paint an incomplete portrait of these maps. Analysis of flux, which is essentially the *in vivo* reaction rate, using labeled metabolites allows the completion of the portrait.^64^ Flux analysis determines the flow of a metabolite through a region in cellular space in a given unit of time. This analysis is central to metabolic engineering that aims to enhance product formation by regulating metabolic pathways. To modify the pathway, it is important to understand the factors that control the pathway. Methods that determine flux and its control provide the knowledge needed to improve the properties of a cell. Such analysis not only considers the genetic control of enzymatic reactions but also captures the environmental effect on the pathways.

For flux analysis of natural products obtained from the secondary metabolism of eukaryotes such as plants, importance has been given to the compartmentalization of reactions. Metabolites and corresponding enzymes are located in different organelles with parallel reactions taking place. A traditional LC-MS-based experiment will quantify such metabolites as moles per unit cell volume. For metabolites that are concentrated within one or more cellular spaces, the determined concentration will be lower than the actual concentration driving the metabolic reaction in the given space within the cell. Further, experiments involving isotopic labeling of metabolites need to consider the possible difference in the labeling patterns of metabolites located within different compartments of the cell. Well-designed flux analysis experiments can help deconvolute the measurements such that distribution of flux corresponding to different pathways leading to the formation of same product can be calculated.^65^

Besides the study of metabolic flux of primary metabolism, the use of stable isotope tracers has been widely applied in the natural product field. Both, for the discovery and structure elucidation of metabolites as well as the investigation of their biosynthetic origin, the use of stable isotope is a powerful tool.^66,67^ With the rise of non-targeted metabolomics and molecular networking strategies, several studies have made use of stable-isotope precursor feeding to identify natural products with specific biosynthetic precursors such as colibactin,^68^ xanthomonic acid^69^ and to investigate the biosynthesis of fungisporin and the discovery of related analogues.^70^ Yet, key drawbacks include metabolic scrambling of precursors, high costs of isotopic studies and the fact that isotopically labeled metabolites are not available for all precursors and metabolites of interest. For such cases, a key advance in stable isotope studies is the inverse stable isotope labeling technique, termed INVERSIL.^71^ With this method, typically used for organism that grow on minimal media with limited carbon and nitrogen source, the organism is cultured with 13C and/or 15N as sole carbon and nitrogen source. If full labeling is achieved, natural precursors are fed with the goal to observe “inverse” mass shifts, from fully isotope labeled metabolites to lower masses for the metabolites that contain the fed natural precursors. Cummings *et al.* used this technique for the discovery and structure elucidation of a set of new homo serine lactones.^71^

### 2.4 Affinity-based metabolomics approaches

Affinity-selection mass spectrometry (AS-MS) is a method that exploits the binding of a compound to a target to select bioactive compounds from a complex pool of small molecules (*e.g.*, a plant extract). This high-throughput method can be used to screen large numbers of compounds,^72,73^ up to 20 000 compounds in one sample have been analyzed so far.^74^ AS-MS methods can provide insights into which compounds in a complex mixture bind a target molecule of interest; however, a key drawback of these methods is the fact that they do not indicate which ligands are activating, inhibiting, or binding without affecting activity. However, given that binding can trigger an effect on a receptor, enzyme, or other target molecule, affinity metabolomics workflows may deliver valuable information about which molecules should be prioritized for further bioactivity tests.

While some AS-MS methods rely on the immobilization of the target others take advantage of non-immobilized or soluble targets. Use of soluble targets is advantageous because it allows for ligand binding at any site and can also detect allosteric effects. Immobilizing the target, as in methods like frontal-affinity chromatography (FAC),^75^ can introduce spatial restrictions or changes in the three-dimensional structure due to the immobilization. Size-exclusion chromatography (SEC) AS-MS, pulsed-ultrafiltration (PUF) AS-MS and magnetic microbead affinity selection MS (MagMASS) are three widely used AS-MS methods that rely on binding of soluble targets. SEC- and PUF-AS-MS first incubate the target molecule with the compound mixture or extract of interest. By choosing the right conditions for the target molecule in the solution (*e.g.*, non-denaturing pH value, temperature, *etc.*), one or more compounds from the mixture can bind the target molecule, either directly at the active site or allosterically.

While PUF-AS-MS shares the same starting point as SEC, it is a one-dimensional method^76^ whereas SEC-AS-MS then uses a two-dimensional chromatographic set-up to separate non-binding molecules and characterize the binding molecules.^77^ In SEC-AS-MS, the target molecule–binder complex can be separated from the smaller non-binders in the first dimension due to its larger hydrodynamic radius. In the second dimension, a reversed-phase method denatures the target molecule due to a comparably high organic solvent composition in the mobile phase, which facilitates dissolution of the target molecule and the binder. This is followed by the separation of several binding molecules. An infusion of the eluate into an MS system facilitates the final identification of the binding molecules. While the PUF-AS-MS workflow is slower, one advantage of this method over the two-dimensional methods is that less advanced LC systems can be utilized. Using this strategy, non-binders are removed by ultrafiltration after the incubation, and the target molecule is denatured to release the binder. This eluate can then be analyzed using RP separation on an LC-MS system. The MagMASS approach differs from PUF-AS- and SEC-AS-MS because the target molecule is immobilized on a magnetic bead before incubation with the compound mixture. After incubation, non-binders can be separated from binders by immobilizing the magnetic beads on the bottom of the reaction vessel and washing, then the target molecule is denatured to release the binders after washing. As with the other AS-MS methods described above, the eluate can be analyzed by LC-MS. However, a key limitation of the MagMASS method is the fact that it is sterically restricted.^78^

(Tandem) affinity purification refers to the use of affinity-based protein purification using tagged proteins and magnetic beads. Use of this strategy was demonstrated by Li *et al.*^79^ for the elucidation of small molecule–protein interactions in yeast. In this study, affinity-based protein purification was performed using magnetic beads alongside proteins tagged with an IgG-binding domain. After washing, the eluate – consisting of small molecules that are binding partners – was analyzed by LC-MS. This technique provides an overview of the binding partners of a protein, but this does not necessarily translate to bioactivity. Therefore, tandem affinity purification can be used to prioritize candidates for bioactivity assessment.

MS binding assays can be combined with AS-MS to provide insights into molecular function. In a classical MS binding assay, a target is incubated with a ligand which is then released and measured. Quantification can be performed without the release of the ligand if the ligand is tagged with an immobilized radioactive compound. These classical MS methods are most commonly used for the investigation of enzyme kinetics; however, Gabriel *et al.* recently combined MS binding assays with affinity-selection MS for an activity-based drug discovery screen.^80,81^ This study exploited structural insights into GAT1 inhibitors gamma aminobutyric acid (GABA) and (*R*)-nipecotic acid to explore the membrane transport protein, GABA transporter type 1 (GAT1). Toward this end, a derivative of nipecotic acid was reacted with a library of aldehydes to form oximes; these oximes were then identified by their tag using multiple reaction monitoring (MRM) MS experiments. The oxime derivatives were subsequently incubated with GAT1 in two experiments. In the first experiment, the total binding of the library compounds was determined, taking both specific and nonspecific binding into account. In the second experiment, GABA, a strong competitive inhibitor of GAT1, was added to the incubation mix to occupy all of the specific binding sites in order to preserve only nonspecific binding (*e.g.*, at the membrane, spatially separated from the native target). By using denaturation of target to liberate ligands in both experiments, determining the compound composition by LC-MS, and subsequently removing nonspecific binders from the total binding experiment, this experimental set-up facilitated the determination of new potential binders.

### 2.5 Native metabolomics

Native mass spectrometry is a technique used to analyze the structure and function of intact proteins and other biomolecules in their native structural form.^73^ To accomplish this, native mass spectrometry is typically run at neutral pH and a given ionic strength. Even as native mass spectrometry was first used in the early 1990s (soon after the invention of electrospray ionization), analyses of intact proteins in native conditions are less evolved than bottom-up, peptide-based approaches.^84^ This is because challenges remain in ionization, detection, and analysis of intact proteins *via* mass spectrometry. Native mass spectrometry has made significant advances over the last decade and has both been applied to analyze intact proteins and protein complexes which have been extensively reviewed.^82,85^

Recently, a modified native mass spectrometry-based set-up was used to probe for metallophores in culture supernatant extracts and complex environmental samples.^86^ This study introduced an experimental approach to specifically probe for the metal-small molecule interaction using mass spectrometry. This experimental approach addressed challenges in finding metal chelating molecules, which can often lose metal coordination at low pH and high organic solvent content used in chromatography and sample preparation.

**Fig. 4:**
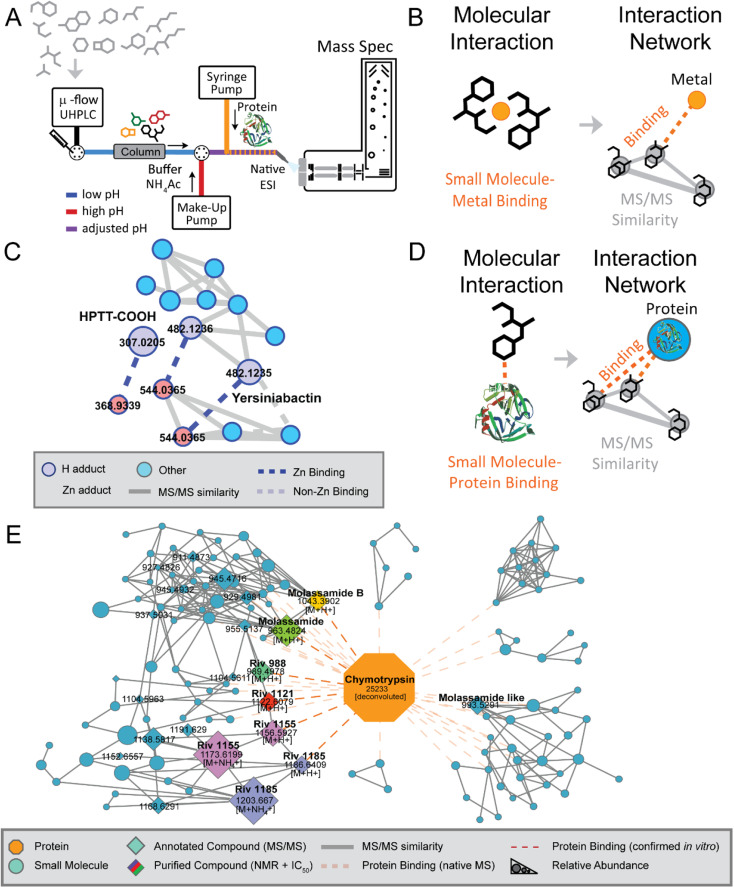
Native MS approach and discovery. (Panel A) A schematic overview of the native metabolomics workflow. This native metabolomics workflow utilizes a microflow UHPLC coupled to a mass spectrometer in addition to a make-up HPLC pump for post-column pH modulation alongside direct infusion of a target protein or metal. (Panel B) Molecular interaction between a small molecule metabolite and directly infused metal salt is revealed using ion identity molecular networking (IIMN). (Panel C) This strategy facilitated the discovery of yersiniabactin–zinc binding along with zinc-binding by the yersiniabactin-truncation HPTT-COOH. (Panel D) Other modification of the method used proteases as binding partner to identify small molecule–protein interactions, which resulted in (Panel E) the discovery of novel chymotrypsin inhibitors. Modified from Reher *et al.*^83^

Previous techniques for elucidating metal-binding have relied on properties of the metal-adduct itself, including the presence of metal-specific isotopic signatures,^87,88^ mass defect/Kendrick analysis,^89^ or analysis of hyperfine splitting isotope patterns.^90^ Yet, these strategies do not work if the metal-coordination is lost. In this study, the mass spectrometer set-up was modified to accommodate direct infusion of metal salts over the entire LC gradient. Simultaneously, the mass spectrometry set-up was also modified to allow for the simultaneous use of two UHPLC pumps, facilitating alteration of pH. In the earliest studies, an external HPLC pump supplying ammonium acetate buffer was “T-ed in” after LC separation at low pH to raise the pH (Fig. 4A and B). This step, which raises pH to any relevant level that researchers hypothesize metal binding occurs at, is essential for mimicking biologically relevant conditions. With this set-up, proof-of-concept experiments were performed to demonstrate this strategy's ability to find known siderophores from *E. coli* Nissle supernatant extracts (yersiniabactin, aerobactin, HPTT-COOH) and from dissolved organic matter samples from the California Current Ecosystem (domoic acid).^86^ Next, researchers were able to use this method to elucidate the novel zinc-binding to yersiniabactin^91^ (Fig. 4C). Additionally, researchers utilized this method to find that the bioactive components of the traditional Samoan medicinal plant (*P. insularum*), rutin and nicotiflorin, are iron-binders.^92^

The native metabolomics strategy has also been applied to probe non-covalent interactions between proteins and small molecules. Here a protein of interest is infused post-column after pH adjustment, while metabolomic samples of interest are analyzed using non-targeted metabolomics methods in consecutive LC-MS/MS runs (Fig. 4D). This approach has been central for the discovery of the rivulariapeptolides, a family of potent chymotrypsin binders from *Rivularia* sp., a marine cyanobacterium collected at Carlos Rosario Beach in Puerto Rico.^83^ The full structures of four new 3-amino-6-hydroxy-2-piperidone (Ahp)-cyclodepsipeptides were determined using *in silico* structure prediction from high resolution MS and MS/MS data along with 1D and 2D NMR analysis (Fig. 4E).

A central tool that enables the data analysis of native metabolomics is ion identity networking,^93^ available for example in MZmine 3, which integrates retention time and chromatographic peak shape correlations into MZmine outputs for molecular networking, was used for metal-binding native metabolomics; similarly, user-defined *m*/*z* offset corresponding to the mass of protein-of-interest can be applied for protein-small molecule native metabolomics. These two examples illustrate how combining new strategies that merge experimental with computational advances can lead to understanding molecular function. We suspect that new technologies for understanding function through modified experimental set-up paired with computational strategies will continue to build on the approaches described above.

### 2.6 Chemical metabolomics

The capacity of a molecule to interact with a specific target is intricately linked to its chemical structure. Therefore, the concepts of functional group and the spatial orientation of the atoms play a pivotal role when determining the affinity of a molecule towards any kind of biological target. Thus, the concept of functional groups is central to research fields such as medicinal chemistry, ecology, or toxicology, among others.^94^ HPLC-MS and HPLC-UV techniques have emerged as the predominant analytical tools for detecting NPs, primarily due to their versatility. However, natural extracts are complex mixtures containing several metabolites, and some natural products are challenging to detect as they may not possess the physicochemical properties required for the detection through the techniques mentioned above. For convenient detection through HPLC-UV-MS, a molecule should be retained on commonly used LC columns, should have a chromophore with characteristic UV-visible light absorbance, and should be ionizable. Various approaches have been developed to selectively increase the detectability of a molecule of interest within a complex matrix. A largely used strategy for the detection of small molecules that are not readily detected by LC-MS is “chemical labelling” (Fig. 3D).^95–97^ This strategy involves the use of probes called derivatization agents (DAs) that possess specific reactivity towards certain functional groups on the analyte molecule. Simultaneously DAs possess the physicochemical properties that the analyte molecule lacks such as the presence of a chromophore, lipophilicity, ionizability, heteroatoms with characteristic isotopic pattern (for instance, Cl, Br), that facilitate detection. The sample mixture is derivatized prior to the HPLC-MS analysis.^21^ Chemical labelling has become a widely employed approach not only in the NPs research, but also in other research fields including proteomics,^98,99^ food-safety,^100,101^ forensics,^102,103^ environmental analysis.^104,105^ To comprehend the significance of this approach within the NPs panorama, it is worth having an overview of the natural chemical space, and on the most frequently encountered functional groups produced by biosynthetic routes. To this end, Ertl and Schuhmann, recently conducted a chemoinformatic investigation of the NPs chemical space.^94^ Their aim was to reveal the most common functional groups occurring in nature, and to distinguish them from the synthetic ones. Over 260 000 NPs from the richest databases (DNP, Natural Product Atlas, TCM Database) were inspected, and 2785 unique functional groups were retrieved. This study revealed that NPs are frequently highly oxidized molecules, in contrast to synthetic products (13 million of structures from the ZINC database),^106^ where highly nitrogenated and chemically more easily accessible functional groups were the most common. Functional groups such as hydroxyl group, phenols, ketones, aldehydes, carboxylic acids, alkenes, dienes and Michael-acceptors, were the most common functional groups among NPs. In the field of NP this methodology has been mainly used in the framework of “targeted metabolomics”, to improve their detection. Derivatization protocols have been developed for functional groups including, but not limited to alcohols, phenols, ketones, aldehydes and carboxylic acids.^96,97^ Hydroxyl group is the most abundant functional group found in NPs. Many chemically diverse NPs, such as paclitaxel, spiramycin, vancomycin, doxorubicin, and kanamycin, contain at least one hydroxyl group. However, molecules containing alcoholic and phenolic functional groups are often very polar and are not efficiently retained on common reversed-phase columns, and they are mainly analyzed under negative polarity on the mass spectrometer.^107^ Therefore, various derivatization strategies have devised for such functional groups. These strategies generally employ strong electrophiles as DAs since the phenolic group is a weak nucleophile and the alcoholic group is even weaker.^21,107^ Anhydrides, acetyl chlorides and benzoyl chlorides have been widely employed for derivatizing molecules containing alcohol and phenol groups.^108,109^ For instance, the use of acetic anhydride aided in the discovery of chlorizidine A, a cytotoxic alkaloid obtained from a marine *Streptomyces* sp. The diacetyl derivative of chlorizidine A has better stability, facilitating convenient isolation.^110^

Ketones, aldehydes, and carboxylic acids are among the frequently encountered functional groups in the landscape of NPs. Carbonyls are derivatized by using nucleophilic agents as the carbonyl group is inherently electrophilic. Hydrazides and hydrazines, especially 2,4-dinitrophenylhydrazine, are commonly used as DAs for this purpose.^111^ Several NPs, such as spiramycin, kitasamycin, and rotenone, contain carbonyl groups, and in some NPs, the carbonyl groups are known to participate in the interaction with a biological target.^21,112^ The carboxyl group is also commonly found in remarkable NPs, an example is penicillin. Amines are usually the first choice for derivatizing carboxyls, and these DAs typically contain an aromatic ring or quaternary nitrogen that improves UV absorption and ionization in positive mode (in contrast to the carboxyl group that is ionized under negative polarity).^21,113^

Several methods have been devised for the detection of analytes containing the amine functional group, particularly primary and secondary amines. 6-Aminoquinolyl-*N*-hydroxysuccinimidyl carbamate (AQC) is probably the most widely employed DA employed to derivatize amines. Amines are commonly found in various classes of NPs, including alkaloids, non-ribosomal peptides (NRPs), and ribosomal peptides (RiPPs). They are also found in anthracyclines, such as the antineoplastic drug doxorubicin, and aminoglycosides such as the antibiotic gentamicin.^112,114,115^

Some derivatization strategies have been developed also for the targeted detection of less common functional groups, such as Michael acceptor systems (*e.g.*, α, β-unsaturated carbonyl) and ring-strained groups (*i.e.*, β-lactone, β-lactam, and epoxide rings). These functional groups usually interact with nucleophilic residues of proteins contained in specific amino acids such as serine, cysteine, lysine, asparagine, arginine, and glutamate. These nucleophilic centers are also present in DNA and other biologically relevant compounds, such as glutathione.^116–121^

Over the years, mimicking this unique reactivity has turned into a strategy to detect these functional groups in a targeted fashion, for this purpose numerous thiol containing chemical probes (*e.g.*, glutathione, mercaptoethanol, 4-chlorothiophenol),^122–124^ have been utilized. Although these approaches have been in certain cases efficiently applied for NP discovery, the practice of labeling functional groups in uncharacterized NP mixtures using a non-targeted metabolomics approach is uncommon in NP discovery, and there are currently only a few existing examples.^124–128^ Conversely, as mentioned earlier, this approach is frequently utilized in a targeted fashion. However, we believe that the structural information obtained through this approach could be implemented into non-targeted analysis and current metabolomics pipelines.

### 2.7 NMR-based metabolomics

In addition to MS, the use of nuclear magnetic resonance (NMR) spectroscopy is widely applied in metabolomics studies, and the two techniques are largely complementary.^129^ In addition to the high reproducibility, speed and non-destructive nature of NMR, its capabilities for *de novo* structure elucidation make NMR an indispensable tool for the structural characterization of unknown molecules. Despite it having a lower sensitivity compared with MS, it is not affected by the presence of salts and can be an absolute quantitative analytical technique. Furthermore, NMR measurements have the advantage of being non-destructive.

NMR spectroscopy is a powerful tool for the structural elucidation of compounds that can also be exploited to investigate the surrounding atoms, which can be leveraged to measure inter-molecular binding affinities. In a classical natural product discovery, molecules have to be collected offline, dried and resuspended in deuterated solvent (for the sake of solvent suppression) for the structure elucidation by NMR. This changed with the dawn of NMR-flow-through cells in 1979;^130^ here complex mixtures can be injected into the NMR without pre-purification, as the HPLC system served as online purification system.

Other experiments, such as (one-dimensional) ^13^C-NMR, ^15^N-NMR or ^19^F-NMR have to be performed offline, as they are time-consuming. Further promising tools for either structural elucidation or affinity profiling of, *e.g.*, small molecules to proteins comprise a large variety of multi-dimensional methods such as TOCSY,^131^ COSY,^132^ NOESY/ROESY,^133^ and HMBC.^134^

Another useful technique for NMR-based metabolomics is solid-state (SS) high resolution magic-angle-spinning (HRMAS)-NMR. This approach uses the spinning of a solid sample (a tissue, a plant or something comparable) at a “magic” angle of 54.74° at a high frequency. In this way, the broadening of the signals is effectively removed, as some broadening-effects, such as dipolar interactions, are reduced.^135^ This facilitates the sample preparation and has been used for the metabolomic screening of, *e.g.*, cancer tissue,^136,137^ metabolite screening in neurons,^138^ metabolic syndrome,^139^ plants^140^ or other. Here, not only the microbiome, but also changes in the microbiome resp. metabolome due to perturbations by drugs, pesticides or other can be detected.^141^

NMR-based metabolomics can be used for the quantification of the metabolomic change in cells perturbed by drug treatments.^142^ Here, mainly cancer cell lines were used and treated with different compounds. Subsequent data analysis led to the identification of four drugs having an impact on a crucial clinical parameter (ratio lactate to pyruvate).^142^ NMR-based metabolomics has also been used for the discovery of important pathways, *e.g.*, in glioma cells.^143^ In this study, the fingerprint of the metabolome of a cancer cell in NMR was used over time for the elucidation of a crucial pathway in the cell. To do so, the complex NMR spectra underwent statistical analysis and were compared to a database. Results showed that the investigated compound increased the Warburg effect and simultaneously blocked glioma-associated oncogene Gli1. This could elucidate a possible target for the treatment of gliomas.^143^

An example of the employment of NMR in functional analysis is the detection of protein–metabolite interactions, in fact NMR spectroscopy has a long-standing history of being used to effectively detect and measure a wide range of protein–ligand interactions,^144–146^ which is also applicable in a high-throughput fashion.

As previously mentioned, metabolomics is often used coupled with genetics to unveil the role of specific genes or proteins. NMR-based metabolomics was also successfully employed in this way to qualify and quantify metabolites arising in response to genetic editing in model organisms. For instance, NMR was employed to investigate the effect of *alr* (alanine racemase) inactivation on *Mycobacterium smegmatis* cellular metabolism, solving a long-debated controversy regarding the role of this enzyme in d-alanine biosynthesis.^147^

Another unique application from the NMR-metabolomics toolbox is real-time NMR. Compared with MS, NMR possesses a distinctive capability to perform non-invasive measurements of distinct metabolite pools *in vivo*. To this end, NMR systems have been employed for quite some time in live-monitoring cancer cells and microorganisms cultures, through the introduction of cutting-edge flow NMR systems.^148,149^ A limiting factor of the utilization of such unique tools is that complex real-time and *in vivo* data require dedicated software to investigate them, however nowadays this surge in data collection surpasses the current capacity to extract and interpret information effectively and computational tools are emerging.^150–152^

## 3. Computational functional metabolomics

Computational approaches serve two main roles in complementing the rich set of analytical techniques and tools for assessing functional metabolomics experimentally. First, computational tools can deconvolve and pinpoint metabolites that contribute to bioactivity of extracts and fractions analyzed in high-throughput experiments. Second, building upon known bioactive metabolites, machine learning strategies aim to predict bioactivity directly from structural information.

With the increasing throughput of non-targeted mass spectrometry analysis in metabolomics, the generation of data has been increasingly commoditized. This presents unique challenges in metabolite prioritization along with an opportunity for discovery of novel bioactive metabolites. In this section, we will review and discuss recent computational advances that aim to ascribe functional characteristics to metabolites and these approaches and their relationship to experimental design.

### 3.1 Computational deconvolution of bioactivity response

The high cost of purification and bioactivity testing of individual molecules in complex extracts makes it impractical to exhaustively screen all observed natural products, especially using classical natural products bioactivity methods (Fig. 3A). Within these complex mixtures, there may exist several molecules that show biological activity in a given assay. However, to pinpoint and prioritize which analytical signals are responsible for bioactivity, we can leverage computational methods of complex mixtures that are analytically measured in specific ways to help deconvolve measurements.

The key insight in these approaches is that the presence of bioactive molecules within a complex mixture will yield a measured bioactivity and that bioactivity will disappear in the absence of the molecule. Given this, the key goal is to identify which molecule out of all in the complex mixture is responsible for the bioactivity. The Compound Activity Mapping approach combines metabolomics measurements from traditional sample fractionation^153^ with image-based screening. To measure bioactivity, each fraction is mixed with a collection of cells to determine a cytological response as a high dimension numerical readout. For each metabolite, the cytological response is averaged from fractions the metabolite appears in to create the consensus cytological response. By clustering the fraction cytological response and metabolite consensus response, the hypothesized responsible analyte for the observed activity is determined. The Bioactive Molecular Networking approach (as described above) computational innovates by leveraging chemical structure in the analysis by clustering molecules with similar structures using tandem mass spectrometry information. This integration with molecular networking, organizes related compounds so that molecules are not treated as single entities, but rather families of related compounds that may share similar bioactivity, resulting in a more focused prioritization of bioactive molecules. Synergistic or antagonistic activity between two or more natural products presents a challenge as the assumption of response of bioactivity and molecule concentration may be broken.^32^ Caesar *et al.*^154^ approached this challenge by the deviation from the expected bioactivity of a known bioactive compound. The authors demonstrated the validation of several synergistic compounds, however, this approach requires *a priori* knowledge of a known active molecule.

The Mass Spectrometry Search Tool (MASST)^155^ can be utilized to add context to molecules detected in a bioactivity experiment. The MASST tool finds all occurrences of a query MS/MS spectrum across all public metabolomics experiments. The authors demonstrated an example of a metabolite differentially expressed within two cohorts of mice and utilized MASST to reveal that this metabolite is also differentially expressed in healthy and non-alcoholic fatty liver disease in humans – even without knowing the molecule's chemical structure. The key experimental context is provided by consistent and comprehensive metadata provided by the Reanalysis of Data User (ReDU)^156^ ecosystem that utilizes controlled vocabularies to crowdsource over 71 K experimental samples.

### 3.2 Computational structure to activity prediction

**Fig. 5:**
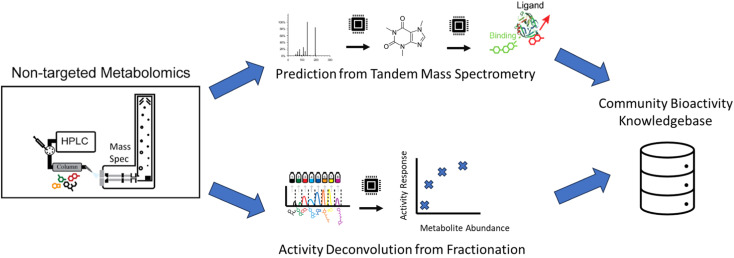
Computational bioactivity prediction. From untargeted metabolomics experiments, two computational strategies try to ascribe bioactivity. First (top) is the direct prediction of activity from tandem mass spectrometry data, and second (bottom) is the computational deconvolution of bioactivity from fractionation experiments. In both approaches, the computational predictions, once validated, should be deposited in a community bioactivity knowledgebase to be used in future analyses and to improve future tools.

A complementary computational strategy to the above deconvolution approaches is to directly predict the functional characteristics of molecules themselves (Fig. 5). Within the chemoinformatics space, there are advances in computationally modeling how small molecules and proteins interact *via* docking.^157–159^ However, the chemical structure must be known to use these strategies. This is a missing component for the vast majority of analytes in complex natural products samples analyzed by mass spectrometry, making it a key limitation of this approach.

One approach is first to transform mass spectrometry data into chemical structural information. In most cases, MS/MS information is utilized. Matching measured MS/MS to libraries^160^ of reference MS/MS spectra with known structures, organized into libraries, is a common approach within the natural products field. Massbank,^161^ Massbank of North America, and GNPS^52^ are key active public MS/MS spectral library resources within the natural products field that include in aggregate 587 213 experimental MS/MS reference spectra. Analog search techniques,^52^ also called hybrid search,^162^ expand on the library search by facilitating the putative identification of new molecules that are related to known natural products.

To bypass the limitations of MS/MS spectral libraries, an alternative computational approach translates MS/MS information directly into structural information as structural fingerprints.^163^ CSI:FingerID^164^ and MIST^165^ utilize machine learning to predict these fingerprints directly from the MS/MS data. CANOPUS^166^ utilizes these predicted fingerprints, which are esoteric to chemists, to predict the natural product class^167^ of the unknown natural product. MSNovelist^168^ takes these fingerprints a step further and predicts structure *de novo*. The resulting structural information can then be utilized to inform activity predictions by computational docking models or natural products chemists. An alternative approach, MS2Prop,^169^ aims to avoid the intermediate step of structure prediction, and rather uses machine learning to predict chemical properties directly from the MS/MS, *e.g.*, drug likeness, synthetic accessibility, *etc.* While promising, all these approaches are fundamentally limited by the information content present within MS/MS spectra, *i.e.*, it is imperative to generate high quality and rich data to leverage these computational approaches.

### 3.3 Capturing knowledge to accelerate computational bioactivity prediction

The availability of public data to help train machine learning models and validate computational methods is needed to improve computational bioactivity prediction. One key data is growing MS/MS spectral libraries within the natural products space by depositing in public resources such as Massbank, Massbank of North America, and GNPS. This effort will enhance the ability of new machine learning models to transform MS/MS data into structural information. This ultimately benefits not only the wider scientific community, but specific labs that contribute by helping ensure that new models will be compatible with a specific experimental setup. Finally, ground truth bioactivity of NPs should be deposited publicly. One of the key aspects is not only capturing known chemical structures associated with bioactivity, but also bioactivity of MS/MS spectra for molecules whose structures have not been solved yet. We hope that such a resource will set the stage for the community to share observations of bioactivity for a vastly larger set of observed analytes and to aid in the development of a new generation of computational tools to aid in the discovery of bioactive compounds.

## 4. Conclusion

In comparison to other fields such as genomics and proteomics, metabolomics (and especially non-targeted metabolomics) is the relative newcomer, yet some consider metabolomics to be the final piece of the “omics” approaches, completing the systematic description of biology.^170^ Non-targeted metabolomics methods have gained significant improvement over the last decade with regards to metabolite coverage, annotation rates as well as standardization of data acquisition, analysis and FAIR data sharing.^171,172^ Thanks to these advances, the field has moved beyond metabolite fingerprinting and the identification of biomarkers, towards gaining mechanistic insights into biological processes.^173^ At the same time, these advances have also been leveraged by the natural product community, where modern non-targeted metabolomics workflow have accelerated the annotation of known molecules and aided the discovery and structure elucidation of novel compounds.^174^

While non-targeted metabolomics has dramatically accelerated natural product discovery, dereplication, and structure elucidation process, several bottlenecks remain. A central problem is still the limited compound identification rate, due to limited spectral library knowledge, our still basic understanding of MS/MS gas phase fragmentation, and sometimes simply due to insufficient fragmentation of small molecules in CID. The gold standard for *de novo* structure elucidation of new compounds is still combined NMR and HR-MS experiments, which require compound purification, typically on the milligram scale. Here, we see a tremendous opportunity for the community to both build and populate larger spectral libraries and to leverage these to better understand MS/MS fragmentation. We anticipate that the constantly growing data will dramatically improve the training of machine learning models, to both better predict MS/MS fragmentation of known chemical structures and propose completely new structures *de novo*. These developments will likely transform both the fields of metabolomics and natural product discovery.

Building on the analytical and technical advancements of the metabolomics field, functional metabolomics approaches will provide scalable mechanistic insights such as metabolite-gene and metabolite–protein interactions. While most functional metabolomics workflows make use of genome-wide metabolite association studies, for example through knock-out strain libraries, new workflows are emerging that directly measure physical interaction and chemical reactions. In this article, we highlighted a range of different approaches, which in our opinion have the potential to transform the field of functional metabolomics and move from correlation and guilt by association-based information to scalable causative biological insights.

For the natural product community, we envision emerging functional metabolomics workflows to be transformative and straightforward to adapt, as the field has a long-standing history for using activity-guided approaches. Obtaining causative functional information at scale will there not only accelerate the understanding of chemical interactions in biology and search for new pharmaceutical lead structures, but also provide critical training data for the development of future machine learning tools.

## 5. Author contributions

All authors performed literature research, wrote, edited, and approved the final manuscript.

## 6. Conflicts of interest

MW is a co-founder of Ometa Labs LLC.
